# Multi-Habitat Radiomics Unravels Distinct Phenotypic Subtypes of Glioblastoma with Clinical and Genomic Significance

**DOI:** 10.3390/cancers12071707

**Published:** 2020-06-27

**Authors:** Seung Won Choi, Hwan-Ho Cho, Harim Koo, Kyung Rae Cho, Karl-Heinz Nenning, Georg Langs, Julia Furtner, Bernhard Baumann, Adelheid Woehrer, Hee Jin Cho, Jason K. Sa, Doo-Sik Kong, Ho Jun Seol, Jung-Il Lee, Do-Hyun Nam, Hyunjin Park

**Affiliations:** 1 Department of Neurosurgery, Sungkyunkwan University, School of Medicine, Samsung Medical Center, Seoul 06351, Korea; seungwon.choi@samsung.com (S.W.C.); krmd.cho@samgsung.com (K.R.C.); doosik.kong@samsung.com (D.-S.K.); hojun.seol@samsung.com (H.J.S.); jilee.lee@samsung.com (J.-I.L.); 2 Department of Electrical and Computer Engineering, Sungkyunkwan University, Suwon 16419, Korea; guraud0810@skku.edu; 3 Centerfor Neuroscience Imaging Research, Institute for Basic Science (IBS), Suwon 16419, Korea; 4 Department of Health Sciences and Technology, Samsung Advanced Institute for Health Sciences and Technology (SAIHST), Sungkyunkwan University, Seoul 06351, Korea; guhalim@skku.edu; 5 Computational Imaging Research Lab, Department of Biomedical Imaging and Image-Guided Therapy, Medical University of Vienna, 1090 Vienna, Austria; karl-heinz.nenning@meduniwien.ac.at (K.-H.N.); georg.langs@meduniwien.ac.at (G.L.); 6 Department of Biomedical Imaging and Image-Guided Therapy, Medical University of Vienna, 1090 Vienna, Austria; julia.furtner@meduniwien.ac.at; 7 Center for Medical Physics and Biomedical Engineering, Medical University of Vienna, 1090 Vienna, Austria; bernhard.baumann@meduniwien.ac.at; 8 Division of Neuropathology and Neurochemistry, Department of Neurology, Medical University of Vienna, 1090 Vienna, Austria; adelheid.woehrer@meduniwien.ac.at; 9 Research Institute for Future Medicine, Samsung Medical Center, Seoul 06351, Korea; heejin1017@gmail.com; 10 Department of Biomedical Sciences, Korea University College of Medicine, Seoul 02841, Korea; js2dark@gmail.com; 11 School of Electronic and Electrical Engineering, Sungkyunkwan University, Suwon 16419, Korea

**Keywords:** glioblastoma, radiomics, biomarker, radiogenomics

## Abstract

We aimed to evaluate the potential of radiomics as an imaging biomarker for glioblastoma (GBM) patients and explore the molecular rationale behind radiomics using a radio-genomics approach. A total of 144 primary GBM patients were included in this study (training cohort). Using multi-parametric MR images, radiomics features were extracted from multi-habitats of the tumor. We applied Cox-LASSO algorithm to build a survival prediction model, which we validated using an independent validation cohort. GBM patients were consensus clustered to reveal inherent phenotypic subtypes. GBM patients were successfully stratified by the radiomics risk score, a weighted sum of radiomics features, corroborating the potential of radiomics as a prognostic biomarker. Using consensus clustering, we identified three distinct subtypes which significantly differed in the prognosis (“heterogenous enhancing”, “rim-enhancing necrotic”, and “cystic” subtypes). Transcriptomic traits enriched in individual subtypes were in accordance with imaging phenotypes summarized by radiomics. For example, rim-enhancing necrotic subtype was well described by radiomics profiling (T2 autocorrelation and flat shape) and highlighted by the inflammatory genomic signatures, which well correlated to its phenotypic peculiarity (necrosis). This study showed that imaging subtypes derived from radiomics successfully recapitulated the genomic underpinnings of GBMs and thereby confirmed the feasibility of radiomics as an imaging biomarker for GBM patients with comprehensible biologic annotation.

## 1. Introduction

Glioblastoma (GBM) is the most lethal primary brain tumor with the median survival of 12–15 months [1]. One of the major obstacles behind this dismal prognosis is the molecular heterogeneity, where GBM is famous for massive inter- and intra-tumor heterogeneity [2,3]. Molecular biomarkers, usually derived from a single confined sub-region of the tumor, are limited to fully representing the biological activities of the entire tumor. Magnetic resonance (MR) imaging is a standard clinical practice for the diagnosis and treatment of GBM patients, which is a safer approach, having no surgical risks. Imaging also has the advantage of integrating information derived from the entire tumor rather than the partial information gleaned from sampling methods [4]. Current guidelines provide limited clinical and biological implications of radiological phenotypes [5,6]. Most radiological features still rely on physicians’ interpretation with a potential subjective bias. Toward data-driven medicine, the need for an objective and quantitative radiographic assessment is increasing. 

Radiomics converts medical images into high-dimensional quantitative data capable of capturing the intra-tumor heterogeneity of the tumor [7]. Previous studies have demonstrated the feasibility of radiomics to predict the prognosis of GBM patients [8,9]. However, as for the molecular correlates of radiomics, a consistent association has yet to be identified and the biologic backgrounds of radiomics remain elusive. 

We reason that phenotypical heterogeneity captured in neuroimaging reflect the underlying biological characteristics [10]. As one study has illustrated [11], classification approach might be more relevant to present the biologic activity of GBM tumors regarding their enormous heterogeneity. Radiomics, as an objective measure of radiographic phenotype, potentially carries the molecular characteristics of tumors and thereby could serve as an imaging surrogate. By integrating genomics and radiomics, we could uncover the biological correlates of an imaging phenotype, thus improving our understanding of molecular underpinnings of radiomics imaging phenotypes.

In this study, we sought to evaluate the feasibility of radiomics as a potent biomarker for GBM patients and explore the transcriptomic traits associated with imaging phenotypes to understand the biological correlates of radiomics. We evaluated the utility of radiomics as an imaging surrogate for GBM patients and identified distinct subtypes using the clustering of the radiomics features. Next, we explored the genomic correlates of these subtypes to establish the genotype–phenotype association. Collectively, we aimed to explore the radiomics-based imaging biomarker for GBM patients with comprehensible biologic annotations.

## 2. Materials and Methods

### 2.1. Study Population—Training and Validation Cohort

We retrospectively screened GBM patients who underwent surgical resection at the Department of Neurosurgery at Samsung Medical Center (SMC) between January 2010 and June 2017. The inclusion criteria were as follows: (i) histologically confirmed primary GBM without prior treatment history, (ii) availability of preoperative magnetic resonance (MR) images taken at SMC with all of four sequences (T1-weighted image [T1]; T1-weighted contrast enhancement [T1CE], T2-weighted [T2], and fluid-attenuated inversion recovery [FLAIR]) with adequate resolution, (iii) contrast-enhancing tumors with measurable volume (≥1 cc), and (iv) availability of clinical records with one or more follow-up visits. In total, 144 patients were finally included. Written informed consent was obtained from all patients, and the Samsung Medical Centre Institutional Review Board approved (IRB no, 2010-04-004, Samsung Medical Institute; 2018-04-023, Samsung Medical Institute) this study. All procedures were carried out following the relevant guidelines. The external validation cohort was collected from the Austrian Brain Tumor Registry network [12]. A total of 56 GBM patients with available MR data were used. Demographic information of the training and external validation cohorts is summarized in Table S1.

### 2.2. MR Image Acquisition

All MR images were obtained using a 3.0-T MR imaging system (Philips). We used four MR sequences: T1, T1CE, T2, and FLAIR. The imaging parameters for each sequence were as follows: T1 and T1CE [Repetition time (TR), 425–500 ms; Echo time (TE), 10 ms; section thickness/gap, 5.0/1.5 mm; matrix, 512 × 512; field of view, 24 cm]; T2 [TR, 3000 ms; TE, 80 ms; section thickness/gap, 5.0/1.5 mm; matrix, 512 × 512; field of view, 24 cm]; FLAIR [TR, 9000–11,000 ms; TE, 125 ms; section thickness/gap, 5.0/1.5 mm; matrix, 512 × 512; field of view, 24 cm]. 

### 2.3. Multi-Habitat Region of Interest (ROI) Segmentation and Feature Extraction

Tumor segmentation was performed on multi-parametric MR images (T1CE and T2WI) by two neurosurgical experts in a semiautomatic manner (SW Choi, MD, five years of experience in neurosurgery; and KR Cho, MD, eight years of experiences in neurosurgery). Multi-habitat regions of the tumors were specified. The contrast-enhancing solid portion was segmented on the axial T1CE images by using the region-growing and thresholding algorithms implemented within the MITK software (www.mitk.org, German Cancer Research Center, Heidelberg, Germany). Similarly, the peri-tumoral edema on T2WI was segmented. The peri-tumoral edema was delineated on the T2 images using the same algorithms. 

To transfer the segmented ROI to other image spaces, image registration using Advanced Normalization Tools (ANTs; http://stnava.github.io/ANTs/) was conducted [13]. For sharing enhancing tumor ROI, T1, T2, and FLAIR images were mapped on to the T1CE space and the enhancing tumor ROI was propagated onto each space using the inverse transformation obtained from ANTs. In the same manner, the ROI of the peri-tumoral edema was transferred from the T2 space.

Radiomics features were calculated using the Python-based open-source software Pyradiomics [14]. Features were calculated in four image sequences (i.e., T1, T1CE, T2, and FLAIR) with two ROIs (enhancing tumor and peritumoral edema region). To quantify the morphological characteristics of the tumor, 14 shape-based features were calculated using enhancing tumor ROI. To measure intensity distribution properties, 18 histogram-based features were calculated using a 128-bin histogram. Twenty-four gray level co-occurrence matrix (GLCM) and 16 gray level size zone matrix (GLSZM) features were computed using 128 and 32 bins for intensity levels to calculate textural information. In total, 478 features were calculated per patient. Details on radiomics features can be found in the Pyradiomics documentation [15].

### 2.4. Radiomics Feature Selection and Development of Radiomics Risk Score

Each radiomics feature of both the training and validation cohorts were z-score normalized based on the mean and standard deviation of the training cohort before further processing. The L1-norm regularized Cox proportional hazard model (Lasso-Cox) [16,17,18] was applied to the training cohort. The optimal penalty weight of the Lasso-Cox model was found in a grid search manner in a 10-fold cross-validation process. The features with non-zero coefficients at optimal penalty weight were finally chosen to build the radiomics risk score.

Radiomics risk score was defined as the output at the initial time point of Cox’s model (i.e., $h\left(X_{i},0\right)$), which was built with the training cohort. The exact formula of the radiomics risk score is as follows:

(1) $$CoxModel=h\left(X_{i},t\right)=h_{0}\left(t\right)\;e^{X_{i}\beta }$$

(2) $$RadiomicsScore_{i}=h\left(X_{i},0\right)=h_{0}\left(0\right)\;e^{\overset{n}{\underset{j=1}{\sum }}X_{ij}*\beta_{j}}$$

where $h\left(X_{i},t\right)$ denotes the hazard of patient i whose feature vector is $X_{i}$ at time t. β denotes regularized coefficients of features and $h_{0}\left(t\right)$ denotes baseline hazard function at time t. The radiomics risk score for the i-th patient is the summation of radiomics features multiplied by the corresponding coefficients derived from Lasso-Cox regression analysis, where n is the number of features selected by LASSO, *β_j_* is the *j*-th weighted coefficient of the selected feature, and *X_ij_* is the *j*-th selected radiomic features for *i*-th patient. This method has been widely used in many radiomics studies encompassing various tumor types [19,20,21,22]. 

Patients with a risk score greater than the median score of the training cohort were assigned to the high-risk group; otherwise, they were assigned to the low-risk group. The survival difference between these subsets was assessed by the Kaplan–Meier plot and log-rank test. For the external validation cohort, radiomics risk score was calculated using the same selected features and the median value of radiomics risk score from the training cohort was used as a cut-off criterion to stratify the patients into two risk groups. 

### 2.5. Discovery and Validation of Radiomics Derived Subtypes

We performed consensus clustering to identify the coherent radiomics subtypes on the training cohort using the selected radiomics features [23]. Consensus clustering is a general, model-independent resampling-based methodology of class discovery and clustering validation [23]. The basic assumption underlying this method is that valid cluster structure could not be influenced by sampling variability, and thus, perturbations of the original data can be simulated by resampling techniques. A consensus matrix is a measure of quantifying the agreement among the clustering runs over the perturbed datasets, which stores, for each pair of items, the proportion of clustering runs where two items are clustered together. Each entry (i,j) of the consensus matrix M is a real number between 0 and 1, and perfect consensus corresponds to M, with all the entries equal to either 0 or 1. Briefly, as we arranged the items accordingly, perfect consensus would be translated into a block-diagonal matrix with non-overlapping blocks of 1s along the diagonal— with each block corresponding to a different cluster—surrounded by 0s. The clustering procedure was repeated with 10,000 bootstraps with 80% of resampling using hierarchical clustering algorithm with Pearson’s correlation distance metric. We determined the optimal number of clusters which yielded the most unambiguous cluster assignments across clustering permutations. This process identified the intrinsic subtypes of GBM tumors defined by radiomics features. 

To validate the radiomics subtypes identified in the training cohort, we trained a prediction analysis for the microarrays (PAM) model by fitting a nearest shrunken centroid classifier on the centroids generated from the clusters of the training cohort [24]. We fitted these centroids in the validation cohort and assigned individual GBM cases to one of the radiomics subtypes defined in the training cohort. 

### 2.6. RNA Sequencing

RNA sequencing libraries were prepared using the Illumina TruSeq RNA Library Preparation Kit v2. The sequenced reads were trimmed and mapped onto hg19 using GSNAP [25]. The resulting aligned reads were summarized into BED files using SAMtools and bedTools (BamToBed version 2.16.2) [26]. The BED files were used to estimate raw counts using the R package “DEGseq” [27]. The obtained raw count values were used in further analyses. 

We performed gene set enrichment analysis (GSEA) to identify the significantly enriched genomic signatures per each radiomics subtypeAll GSEAs were performed using GenePattern from the Broad Institute (http://software.broadinstitute.org/gsea). Next, to remove the redundancy of the GSEA results, we applied Cytoscape for visualizing the enriched genomic signatures [28]. Genesets which exhibited the nominal *p*-value < 0.01 were finally selected to construct the network visualization. Node size inversely correlates with the nominal *p*-values and the node color implies the normalized enrichment score (NES); red color signifies up-regulation, while blue signifies the down-regulation of the corresponding node. Single-sample GSEA (ssGSEA) score was computed for every case using the genesets identified as being significantly highlighted in radiomics subtypes. Z-normalized ssGSEA score was utilized to illustrate a heatmap representing the transcriptomic landscape of GBM patients. 

### 2.7. Statistical Analysis

All statistical analyses were performed using R version 3.6.3 (http://www.R-project.org) [29] and Statistics and Machine Learning Toolbox in MATLAB (The MathWorks, Natick, MA). For the comparison of clinical characteristics, values are presented as the mean ± standard deviation (SD). Continuous variables were compared using the independent sample *t*-test or the two-sided Wilcox test, and categorical variables were tested using the chi-square test or Fisher’s exact test. When comparing the continuous variables with multiple categories (more than three categories), ANOVA or the Kruskal–Wallis test was used where appropriate. Survival analysis was performed using a Kaplan–Meier plot, and the log-rank test was used to show statistical differences between survival curves. 

*p* < 0.05 was used as a threshold for statistical significance. p values derived from multiple comparisons were appropriately corrected by Bonferroni and false discovery rate (FDR) methods [30].

## 3. Results

### 3.1. Radiomics Signature as a Surrogate for the Prognosis of GBM Patients

**Figure 1:**
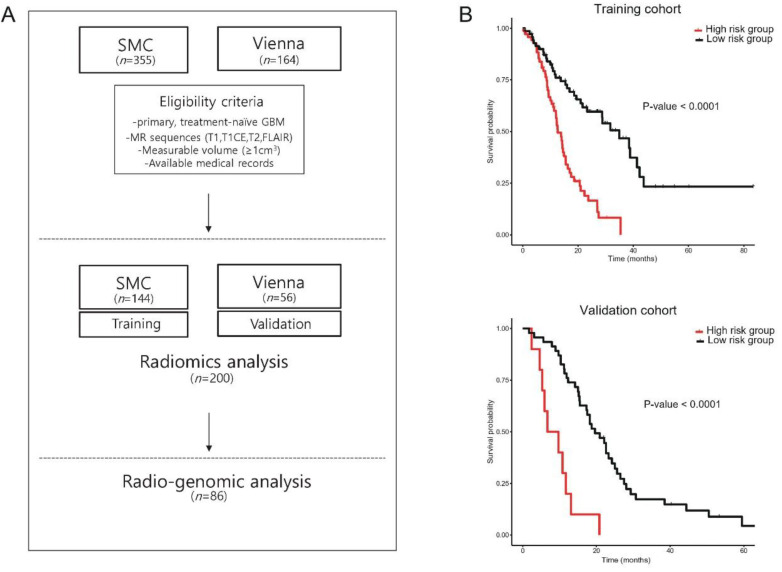
Radiomics workflow. The feasibility of radiomics as an imaging biomarker for glioblastoma (GBM) patients was evaluated through following study platform. (**A**) Schematic illustration of radiomics flowchart in present study; (**B**) survival plots of GBM patients stratified by radiomics risk score, a weighted sum of selected features (above, training cohort; below, validation cohort).

We calculated radiomics features from the contrast-enhancing tumor and peri-tumoral edema regions. A total of 478 radiomics features were extracted from multi-parametric MR images (T1, T1CE, T2, and FLAIR). The overall study flow is summarized in a schematic illustration (Figure 1A). A total of 144 were histogram-derived features and 14 were descriptions for the shape of tumors. The majority of radiomics features are texture-related, which incorporates multiple methods (GLCM and GLSZM) to calculate the textural statistics.

We applied the Lasso-Cox proportional hazard model to the training cohort and seven radiomics features were finally selected as the most relevant features to survival outcome. Among them, one feature was the flatness of the enhancing tumor core, which signified the degree of tumor elongation. Two features were histogram-derived features, which depicted the distribution pattern of voxels of distinct gray-levels. The other four features were texture features reflecting intra-tumor heterogeneity. Of note, no features were selected from the peri-tumoral edema. Detailed information on selected features is shown in Table 1. 

To validate the potential of radiomics as a prognostic biomarker, we defined the radiomics risk score and stratified the GBM patients according to the median value of the score. In the training cohort, the high-risk group demonstrated significantly poor outcomes compared to the low-risk group, and the hazard ratio was 3.56 (Figure. 1B, 95% CI, 2.24–5.65, *p* value < 0.001). This finding was also confirmed in the external validation cohort with a hazard ratio of 19.17 (Figure. 1B, 95% CI, 5.48–67.07, *p* value < 0.001).

### 3.2. Clustering GBM Patients Using Radiomics Features Reveals Distinct Subtypes with Prognostic Significance

**Figure 2:**
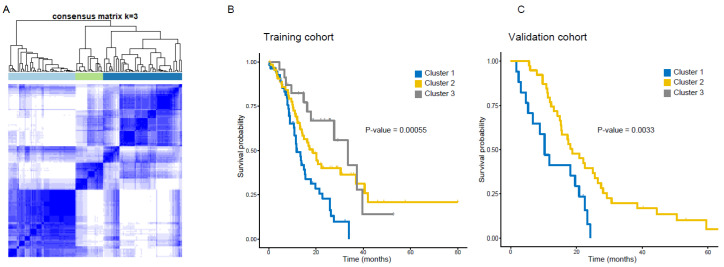
Clustering analysis of GBM patient using radiomics; consensus clustering of GBM tumors with prognostically relevant radiomics features revealed three distinct radiomics subtypes which differed in survival outcome. (**A**) A consensus matrix derived from consensus clustering; each cell of the matrix refers the proportion of clustering runs where two instances are clustered together, ranged between 0 (white) and 1 (blue).1 implies perfect consensus among the entire resampling runs; (**B**) survival plots of GBM patients classified by radiomics subtypes (training cohort); (**C**) survival plots of GBM patients classified by radiomics subtypes (validation cohort).

To identify the imaging prognostic biomarkers, we clustered the GBM patients using the selected radiomics features. According to the consensus clustering, the number of clusters, k, was chosen to be 3 as an optimal solution (Figure 2A, Figure S1) [23]. The three-cluster solution produced the largest k while maximizing the consensus within the cluster and minimizing the rate of ambiguity in cluster assignment across 10,000 iterations. This resulted in 57 patients in Cluster 1 (38.8%), 67 patients in Cluster 2 (45.6%), and 23 patients in Cluster 3(15.6%).

Accordingly, these three GBM subtypes showed distinct survival outcomes as well as radiomics profiling (Figure 2B, *p*-value < 0.001, log-rank test). To validate the clustering subgroups identified in our training cohort, we applied the prediction analysis for microarrays (PAM) to assign each sample in the validation cohort to one of the clusters [24]. This assigned 17 patients in Cluster 1 (30.4%) and 39 patients to Cluster 2 (69.6%). Unfortunately, there were no assigned patients to Cluster 3 in the validation cohort. These subgroups of the validation cohort also exhibited a significantly different survival outcome (Figure 2C, *p*-value = 0.003, log-rank test).

### 3.3. Radiomic Subtypes as Phenotypical Surrogates for GBM Patients

**Figure 3:**
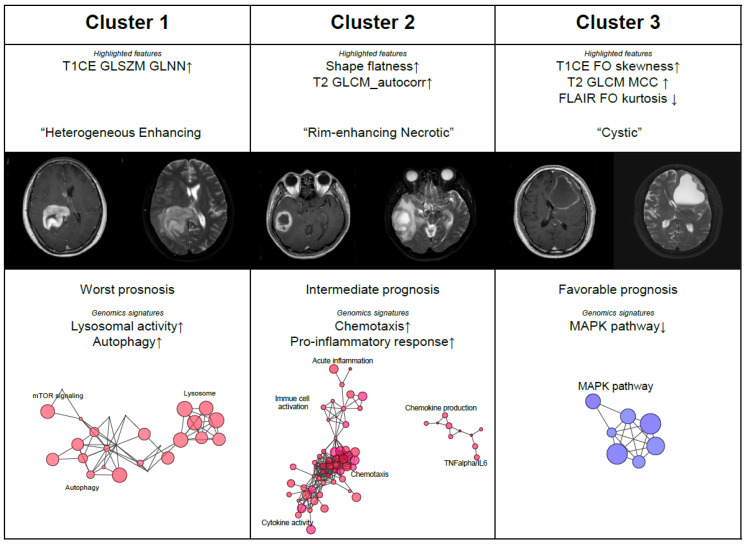
Radiomics subtypes present the distinguished imaging protypes of GBM tumors, which correlated well with genomic signatures. Quantitative radiomics features account for the gross finding of tumors on magnetic resonance (MR) images. Representative MR images as well as enriched genomic signatures of each radiomics subtype were presented.

We identified that GBM patients could be classified by radiomics profiling and these subtypes significantly differed in prognosis. As radiomics features are mostly derived from texture-related statistics, a suitable clinical annotation for radiomics profiling should be clarified. Therefore, we explored the most highlighted features per the corresponding subtype and organized the composition of radiomics profiling with clinically understandable annotations (Figure 3, Figures S2 and S3).

Cluster 1, which exhibited the worst prognosis, is characterized by high values of T1CE-derived gray-level non-uniformity, and normalized (GLNN, texture, GLSZM) features. This texture feature measures the variability of gray-level intensity values in the image, with a higher value indicating the massive heterogeneity in signal intensity. Gross morphology of these tumors (contrast-enhancing solid mass with a mixture of necrosis within the core) were in accordance with high values of T1CE GLNN feature, and we named this cluster as the “heterogeneous enhancing” subtype. 

Tumors in Cluster 2 (“rim-enhancing necrotic” subtype) were characterized by high values of flatness (shape) and T2 autocorrelation (texture, GLCM), indicating the spherical shape with homogenous T2 signal intensity within the tumor. These tumors showed rim-enhancement with a necrotic core, a typical phenotype observed in GBMs. Autocorrelation of GLCM measures the linear dependency of gray-levels in neighboring pixels and thus reflects the intensity heterogeneity. A larger value of T2 autocorrelation implies a tumor with less heterogeneity in T2 signal intensity, which correlated well with the considerable portion of necrotic core observed in Cluster 2 tumors.

Cluster 3 tumors exhibited the most favorable prognosis and were characterized by low values of T1CE skewness and high values of FLAIR kurtosis. Interestingly, this subset also exhibited high values of T2 maximal correlation coefficient (MCC), which indicates the complexity of the texture. Grossly, tumors in Cluster 3 were cystic tumors with thin rim-enhancement on T1CE image and thus were named as the “cystic” subtype. 

Next, we compared the known confounding factors associated with the prognoses of GBM between different radiomics subtypes to validate the potential of radiomics as a prognostic biomarker [31,32,33,34]. None of the known clinical or molecular factors showed significant differences among these subtypes (Table 2). Multi-variate cox regression analysis also supported the radiomics subtype as an independent prognostic factor (Figure S4).

### 3.4. Genomic Correlates of Radiomics Subtypes of GBM 

To comprehend the biologic rationale behind the radiomics, we explored the genomic characteristics of radiomics-defined GBM subtypes. Geneset enrichment analysis (GSEA) was performed to identify the genomic signatures enriched in each individual radiomics subtype. We also searched the correlative gene sets to each radiomics feature to account for the biologic implication of radiomics feature. 

Cluster 1 (the “heterogenous enhancing” subtype) was highlighted by several genomic signatures associated with lysosomal activity and autophagy, as illustrated in Figure 3. Cluster 2 (the “rim-enhancing necrotic” subtype) was distinguished by genomic signatures associated with chemotaxis and pro-inflammatory response. Regarding the correlative gene sets to the T2 autocorrelation feature (Figure S5), this finding is consistent with the radiomics profiling of Cluster 2, where T2 autocorrelation was the most distinguishing feature. Cluster 2 tumors were remarkable with the necrotic core, and thus, genomic signatures associated with chemotaxis and inflammation, which induced by necrosis [35], were in accordance with such findings. Tumors in Cluster 3 (the “cystic” subtype) showed decreased activity in MAPK pathway, one of the most frequently altered pathways in GBMs, suggesting the less aggressive clinical course of these tumors. A heatmap depicting characteristic genomic signatures identified in the present study is illustrated in Figure S6 with supporting GSEA enrichment plots. 

## 4. Discussion

Herein, we demonstrated the efficacy of utilizing radiomics as a prognostic biomarker for GBM patients. Radiomics risk score successfully stratified the GBM patients according to survival outcome and clustering analysis using radiomics features revealed that GBMs were composed of three image-defined subtypes which differed in prognosis. To comprehend the biologic rationale of radiomics as an imaging biomarker, we explored the molecular characteristics of these radiomics subtypes. This approach enabled us to discover potential connections between radiomics and specific genomic traits with relevant clinical translation. 

In the radiomics risk score building step, seven features were identified to be significantly associated with GBM prognosis (Table 1). There was no dominant imaging modality. Two features were derived from T1CE, two from T2, and the remaining from FLAIR. This implies that the comprehensive integration of imaging modalities is required for the prognostication of GBM patients. In terms of features’ category, texture features of GLCM and GLSZM were heavily involved in radiomics risk scores. Previous studies have shown that intra-tumoral heterogeneity measured with texture features is a strong prognostic factor for tumor behavior [36,37]. Moreover, the risk score was sensitive to the flatness shape feature of the tumor. This corroborates many studies reporting poor prognosis where tumors show an irregular or spatially distorted shape [38,39]. We conducted a sensitivity analysis on our Cox regression model in a one-at-a-time manner to interpret the model further (Table S2.).

Cluster 1 tumors (the “heterogeneous enhancing” subtype) were characterized by high values of T1CE GLSZM GLNN feature, which indicating the high variability of signal intensity on T1CE image. This radiomic profiling accounts for the gross morphology of Cluster 1 tumors, which exhibited intermingled necrosis within the tumor. Interestingly, genomic signatures associated with lysosomal activity and autophagy were enriched in Cluster 1 GBMs. A lysosome is an important recycling organelle associated with autophagy, and also functions as a relay hub for signaling pathways driven by the mechanistic target of rapamycin complex 1 (mTORC1) [40,41]. Although autophagy is known to have dual functions in respect to tumorigenesis [42], its pro-tumorigenic role inducing the treatment resistance against temozolomide (TMZ) is more emphasized in GBM studies [43,44,45]. Such molecular traits are in accordance with the worst prognostic implication of Cluster 1 tumors. Additionally, the inverse correlation between autophagy activation and necrosis may account for the gross morphology of limited extent of necrosis observed in these tumors. 

The radiographic presentation of Cluster 2 (the “rim-enhancing necrotic” subtype) is the prototype of GBMs, with rim-enhancing spherical tumors with a necrotic core. This morphology is well explained by the highlighted features in Cluster 2 (T2 autocorrelation and flat shape feature). Necrosis is the hallmark of GBMs and is known to induce inflammation in the surrounding tissue [35]. In accordance with the remarkable findings regarding these tumors (necrotic core), genomic signatures related to chemotaxis and pro-inflammatory response were significantly enriched in Cluster 2 tumors. Of note, the TNF-alpha pathway was highly upregulated in these tumors, suggesting the necrotic milieu of GBMs [35,46].

Cluster 3 (the “cystic” subtype) comprised a minor subset of GBM patients in the training cohort. We could not identify the patients assigned to this cluster in the validation cohort. These tumors grossly exhibited cystic morphology with thin rim-enhancement. The favorable clinical outcome in cystic GBMs has been previously described [47,48,49], and accordingly, patients in Cluster 3 also showed the best prognosis. Downregulation of the MAPK pathway, which is usually upregulated in the majority of GBMs, indicating the aggressiveness of tumors [34], was correlated to the indolent clinical course of patients in this subtype.

Previously, Itakura et al. identified three distinct phenotypical subtypes of GBM patients using T1CE MR images [50]. These subtypes are named as “pre-multifocal”, “spherical”, and “rim-enhancing”, respectively. The phenotypical characteristics of these subtypes are similar to those of ours; for example, the “spherical” subtype corresponds to Cluster 2 (“rim-enhancing necrotic” subtype) in our study, which is characterized by the spherical shape with high values of the “flat” feature. Meanwhile, the “rim-enhancing” subtype, marked by central hypo-intensity on T1CE image, is consistent with Cluster 3 (“cystic” subtype), presenting the cystic morphology of which with thin rim-enhancement. As we utilized multi-parametric MR images including T2 and FLAIR, the subtypes defined here were supplemented by more detailed descriptors, which might have been missed in previous studies. It is noteworthy that despite different sets of image-derived features and methods of clustering, consistent phenotypical subtypes have been presented across the studies. Moreover, their prognostic implications have been maintained invariably, regardless of the study scheme. 

These reproducible phenotypical subtypes signify the potential of imaging biomarkers. Importantly, they reflect distinct molecular underpinnings and their distinction may imply the different therapeutic vulnerability. For example, treatment strategy targeting autophagy would be more effective against Cluster 1 tumors, whereas a hostile tumor microenvironment would be a more idealistic target for Cluster 2 tumors considering their predominant inflammatory milieu.

Autophagy induction has been implicated in the response to TMZ for malignant gliomas [51]. Autophagy inhibition has been shown to promote the sensitivity towards TMZ in glioma stem cells [44]. Several agents modulating autophagy have been introduced [52] and some of them, including chloroquine, have already shown some beneficial effects in malignant glioma patients [43]. Imaging phenotypes defined by radiomics subtype may enable to identify the best subgroups that would derive therapeutic benefit from these agents. 

Tumor microenvironment (TME) is a key target for next-generation therapeutics for gliomas. Despite flourishment of immunotherapies, GBMs remain immune-desert tumors and thus, modulating the immuno-suppressive TME is the priority to overcome their innate treatment resistance against immunotherapy [53]. The inter-relationship between tumor and immune cells has been emphasized in promoting the malignant potential of GBMs as well as treatment resistance; M1 polarization of macrophage was identified as a potent mechanism of how GBMs were eradicated following triple combination of oncolytic virus and immune checkpoint inhibitors [54] Chemotaxis and proinflammatory milieu of Cluster 2 tumors are not synonymous to immune-inflamed TME; however, detailed immunophenotyping may account for the exact implication of these genomic signatures in the context of immune landscape. 

Imaging biomarkers are more versatile than molecular biomarkers considering several key challenges against GBMs; they are non-invasive and free from sampling bias, thus reflecting the characteristics of the whole tumor. However, they also have some inherent weakness; data reproducibility and objectivity are the main concerns accompanied by radiographic assessment. Radiomics enabled us to transform the descriptive neuroimaging features into the format of quantitative and objective data, thus contributing the evolution of image-driven data science. Another challenge is their translational ability in terms of biological activity. Many have attempted to annotate the imaging phenotype with understandable molecular correlates; however, no robust association has yet been elucidated. Radiomics is high-dimensional and thus, identifying specific molecular correlates per individual feature would be spurious. Classification analysis is a more applicable approach to explore the molecular rationale behind the radiomics, as shown in present study. 

This study has several limitations; we retrospectively screened patients and thus, selection bias was inevitable due to the inherent nature of the study design. The number of patients was limited, especially for the validation cohort, where we could not identify Cluster 3. The most significant drawback in this study was the lack of experimental validation. The causal relationship between the highlighted genomic signatures and their corresponding radiomics subtype could be validated through well-designed experiments. We can confirm the therapeutic implication of these image-defined subtypes following experimental validation. As a research field, radiomics is still developing, and thus, further studies focusing on the above limitation are warranted.

In conclusion, by assessing the properties of the entire tumor, radiomics may have the potential to provide accurate predictions of patient prognoses. This is a major advantage and may overcome the limitation of molecular biomarkers that draw information from a partial region in the tumor, which is a significant drawback regarding the molecular heterogeneity of GBMs. In the present study, we captured the potential genotype–phenotype association of GBM tumors in terms of radiomics, thereby presenting phenotypic subtypes of GBMs with distinct genomic traits. We applied multi-parametric MR images and multi-habitat ROIs to represent the characteristics of real tumors with sufficient supplements. Radiomics subtypes could serve as imaging biomarkers for prognosis prediction as well as therapeutic strategies. Importantly, these subtypes, much improved in detailed description, are reproducible across studies, which signifies the robustness of phenotypic surrogates for GBM tumors. By integrated radio-genomics approach, we confirmed the potential of radiomics as imaging biomarker for GBMs regarding clinical and genomic significance. 

## 5. Conclusions

The present study has demonstrated the potential of radiomics, an objective and quantitative measure of radiographic phenotype, as an imaging biomarker for the prognosis of GBM patients. Despite previous efforts, biological correlates of imaging phenotype remain elusive. By radiomics, we identified three phenotypic subtypes of GBM tumors, and these subtypes demonstrated distinct prognostic implications as well as genomic characteristics consistent with the prior literature. We showed the molecular correlates of the imaging derived phenotypic subtypes with clinically comprehensible annotations. This approach unraveled the molecular underpinning behind the imaging derived phenotypes, thus supporting the potential of radiomics as an imaging surrogate for GBM tumors. 

## Figures and Tables

**Table 1:** Selected radiomics features associated with prognosis of glioblastoma patients.

Feature Name	Cox-Lasso Coefficient
Tumor shape – flatness	−0.1423
Tumor histogram- skewness (T1CE)	−0.0801
Tumor GLSZM- gray level non-uniformity, normalized (T1CE)	0.0458
Tumor GLCM-autocorrelation (T2)	−0.0905
Tumor GLCM-MCC (T2)	−0.0943
Tumor histogram-kurtosis (FLAIR)	−0.0034
Tumor GLCM-difference entropy (FLAIR)	0.0106

Abbreviation T1CE, T1 contrast-enhancement image; GLSZM, gray-level size zone matrix; GLCM, gray-level co-occurrence; MCC, maximal correlation coefficient; FLAIR, fluid attenuated inversion recovery image.

**Table 2:** Clinical and molecular characteristics of radiomics-defined subtypes.

	Cluster 1	Cluster 2	Cluster 3	*p*-value
	Training cohort			
No. of patients (No. of patients with WTS data)	57 (34)	67 (39)	23 (13)	
Age (years)	57.6	59.8	52.3	0.277 *
Sex (male (%))	56.1 (32/57)	53.7 (36/67)	52.2% (12/23)	0.926 **
pMGMT methylation (methylation (%))	42.1 (24/57)	53.0 (35/66)	59.1 (13/22)	0.314 **
IDH1 mutation (mutant (%))	1.9 (2/55)	3.2 (2/64)	10.5 (2/21)	0.223 **
Operation extent (GTR (%))	45.6 (26/57)	59.7 (40/67)	60.9 (14/23)	0.234 **
	Validation cohort			
No. of patients (No. of patients with WTS data)	17 (0)	39 (0)	0	
Age (years)	58.1	56.4	NA	0.648 ***
Sex (male (%))	70.6 (12/17)	61.5 (24/39)	NA	0.561 **
pMGMT methylation (methylation (%))	NA	NA	NA	NA
IDH1 mutation (mutant (%))	0 (0/11)	10 (3/30)	NA	0.551 **
Operation extent (GTR (%))	42.9 (3/7)	47.8 (11/23)	NA	1 **

* ANOVA; ** Fisher’s exact test; *** Kruskal–Wallis test; Abbreviations: No, number; NA, not applicable; WTS, whole transcriptome sequencing; pMGMT, MGMT promoter; GTR, gross total resection.

## Supplementary Materials

The following are available online at https://www.mdpi.com/2072-6694/12/7/1707/s1, Figure S1: Decision of number of clusters (k) during consensus clustering, Figure S2: Principal component analysis of radiomics profiling of glioblastoma patients revealed the highlighted features per corresponding radiomics subtype, Figure S3: Comparison of radiomics feature values between radiomics subtypes, Figure S4: Hazard ratio plot derived from multi-variate cox regression analysis, Figure S5: Top 30 correlative gene sets to T2 autocorrelation (GLCM) feature, Figure S6: Characterized genomic signatures according to radiomics subtype, Table S1: Molecular and clinical characteristics of study cohorts. Table S2: Sensitivity analysis of the Cox regression model
