# Supplementary material for: Multi-Habitat Radiomics Unravels Distinct Phenotypic Subtypes of Glioblastoma with Clinical and Genomic Significance
Source: Cancers (Basel). 2020 Jun 27;12(7):1707. doi: 10.3390/cancers12071707 (PMC7408408; doi:10.3390/cancers12071707)

Supplementary Material

1. **Supplementary Tables**

2. **Supplementary Figures**

Supplementary Table S1. Molecular and clinical characteristics of study cohorts

	Training cohort (SMC)	Validation cohort (Vienna)	p-value
No. of patients	144	56	
Age (mean±s.d.)	57.8±1.0	56.9±1.8	0.683
Male : Female	78:66	36:20	0.207
IDH1 mutation status (mutant (%))	3.8% (5/131)	7.3% (3/41)	0.397
MGMT methylation status (methylated (%))	50% (71/142)	NA	NA
Operation extent (GTR (%))	54.2% (78/144)	46.7% (14/30)	0.683

*fisher's exact test for categorical value; wilcox.test for continuous value

NA, not available

Supplementary Table S2. Sensitivity analysis of the Cox regression model

Features	Z-score		
	Minimum	Maximum	Cut off for low-risk group

shape – flatness	-3.4472	1.9706	> -0.0976
histogram- skewness (T1CE)	-2.0563	4.4311	> -0.1735
GLSZM- gray level non-uniformity, normalized (T1CE)	-3.0759	3.6433	< 0.3032
GLCM-autocorrelation (T2)	-1.991	3.0934	> -0.1535
GLCM-MCC (T2)	-1.9868	2.9021	> -0.1474
histogram-kurtosis (FLAIR)	-1.1041	5.2424	> -4.1387
GLCM-difference entropy (FLAIR)	-5.6988	1.9248	< 1.3061
	Raw value		
	Minimum	Maximum	Cut off for low-risk group
shape – flatness	0.1784	0.8799	> 0.6121
histogram- skewness (T1CE)	-1.3833	2.8322	> -0.1599
GLSZM- gray level non-uniformity, normalized (T1CE)	0.0389	0.0880	< 0.0636
GLCM-autocorrelation (T2)	1565.5680	9858.9461	> 4562.7913
GLCM-MCC (T2)	0.4151	0.9682	> 0.6232
histogram-kurtosis (FLAIR)	1.9193	14.5936	> -4.1410
GLCM-difference entropy (FLAIR)	2.6083	5.4509	< 5.2202

Abbreviation: *T1CE*, T1 contrast-enhancement; *GLSZM*, gray-level size zone matrix; *GLCM*, gray-level co-occurrence matrix; *MCC*, maximal correlation coefficient; *FLAIR*, fluid attenuated inversion recovery

Supplementary Figure legends

Supplementary Figure S1. Decision of number of clusters (k) during consensus clustering; to determine the optimal number of clusters in training cohort, CDFs (cumulative distribution of consensus matrices) and delta area (area under the CDFs) were evaluated to quantify the concentration of the consensus distribution. The number of clusters, k, was chosen to maximize the concentration by inspecting CDFs' shape and progression.

Supplementary Figure S2. Principal component analysis of radiomics profiling of glioblastoma (GBM) patients revealed the highlighted features per corresponding radiomics subtype

Supplementary Figure S3. Comparison of radiomics features values between radiomics subtypes

Supplemnetary Figure S4. Hazard ratio plot derived from multi-variate cox regression analysis

Supplementary Figure S5. Top 30 correlative gene sets to T2 autocorrelation (GLCM) feature

Supplementary Figure S6. Characterized genomic signatures according to radiomics subtype; a heatmap using single sample geneset enrichment scores (left) and GSEA enrichment plots (right)

Supplementary Figure S1. Survival curves of validation cohort stratified by radiomics subtype

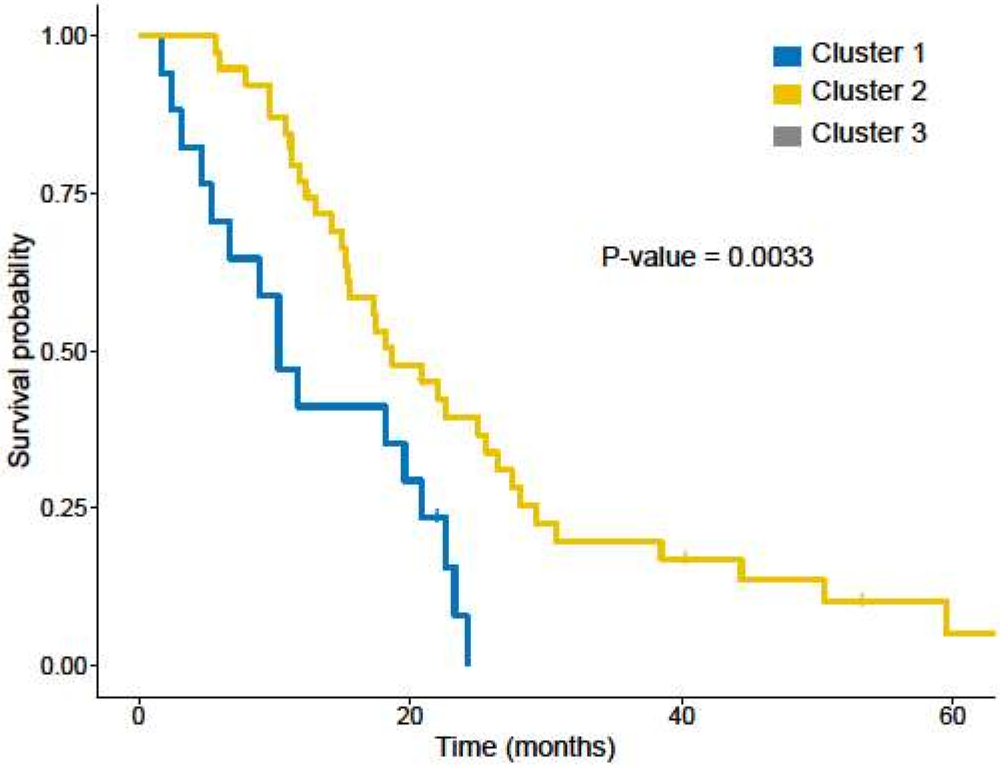

Supplementary Figure S2. Principal component analysis of radiomics profiling of glioblastoma (GBM) patients revealed the highlighted features per corresponding radiomics subtype

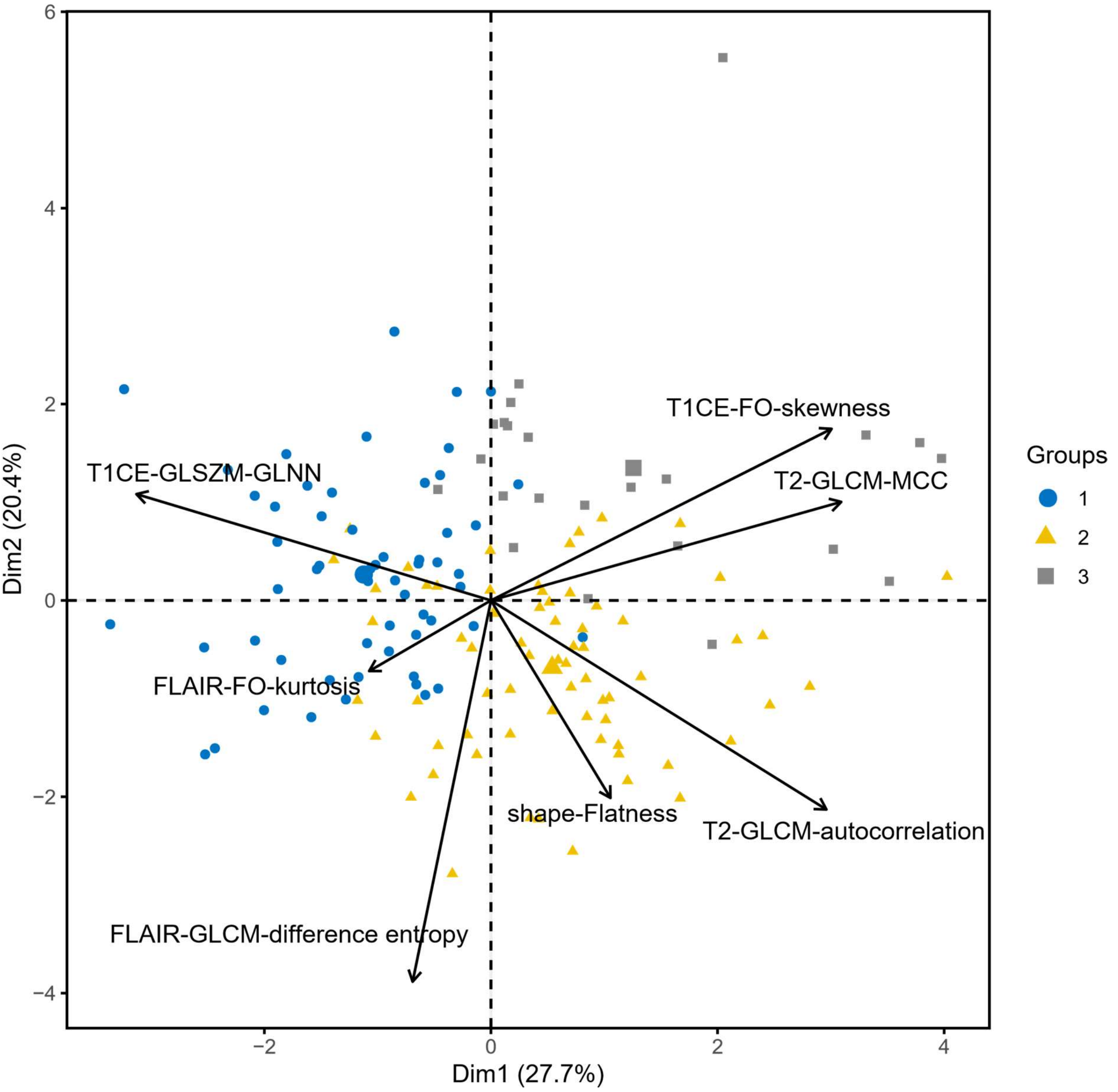

Supplementary Figure S3. Comparison of radiomics features values between radiomics subtypes

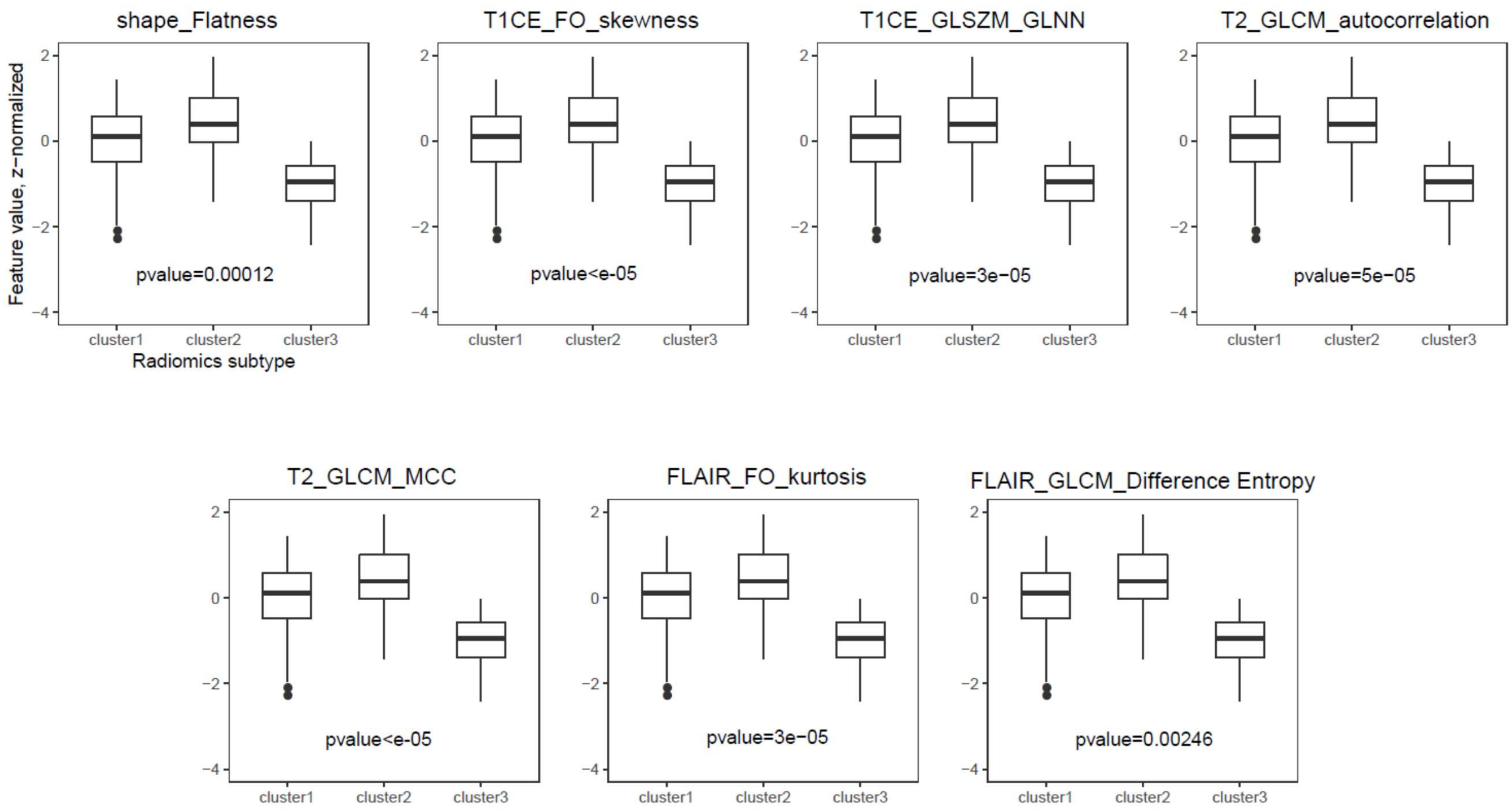

Supplementary Figure S4. Hazard ratio plot derived from multi-variate cox regression analysis

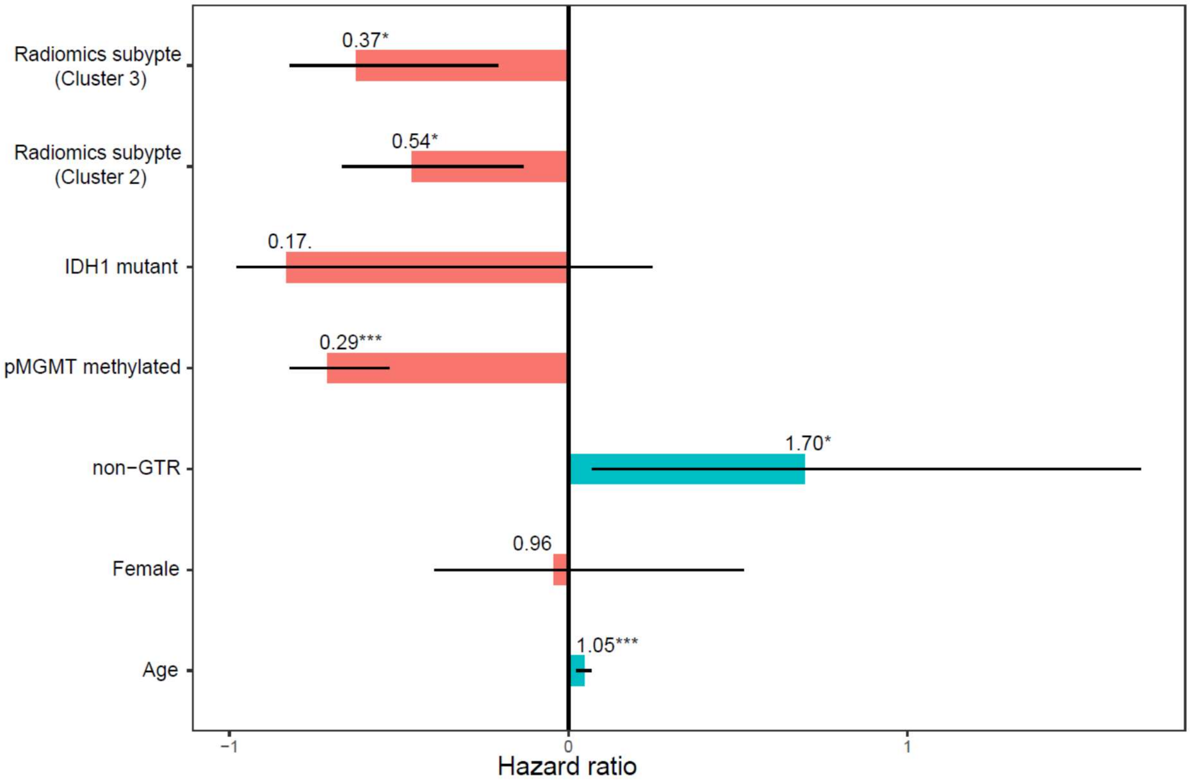

Supplementary Figure S5. Top 30 correlative gene sets to T2 autocorrelation (GLCM) feature

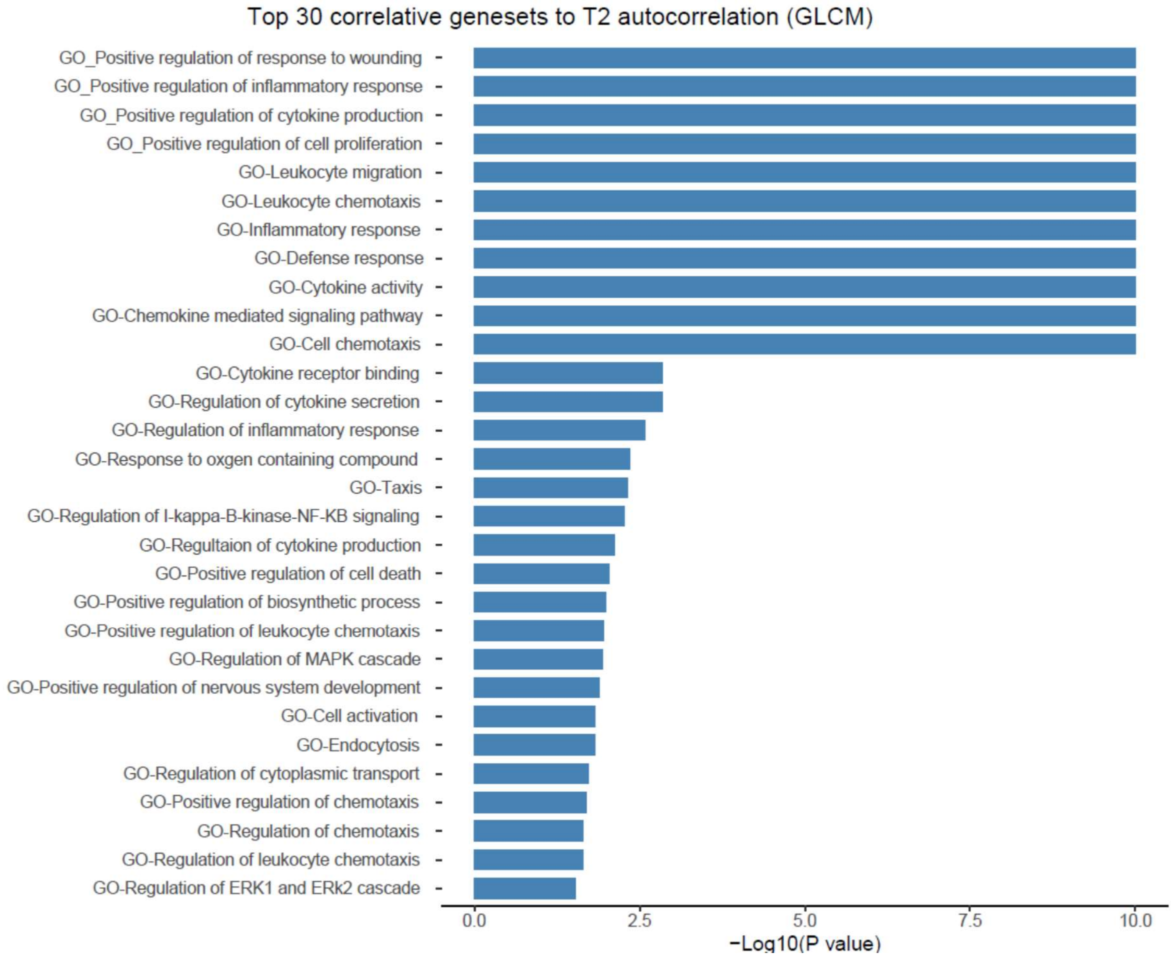

Supplementary Figure S6. Characterized genomic signatures according to radiomics subtype; a heatmap using single sample geneset enrichment scores (left) and GSEA enrichment plots (right)

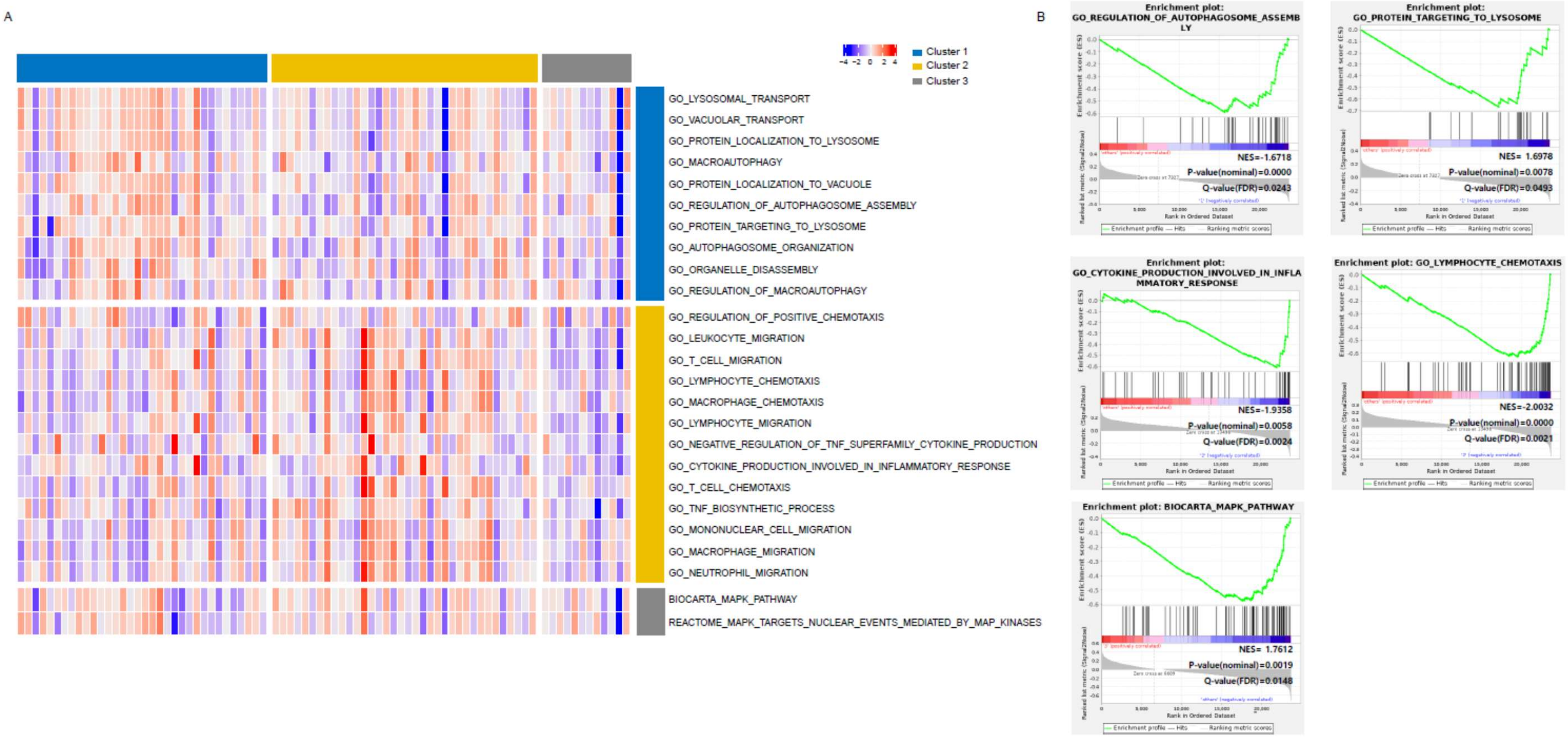

Supplement: Supplementary file 1 [file cancers-12-01707-s001.pdf]
